# Peritonitis on sigmoidal perforation in a cocaine user: A rare case report

**DOI:** 10.1016/j.ijscr.2024.109287

**Published:** 2024-01-20

**Authors:** Mohamed Yassine Mabrouk, Abdelali Guellil, Soussan Haitam, Tarik Deflaoui, Rachid Jabi, Mohammed Bouziane

**Affiliations:** Department of General Surgery, Mohamed VI University Hospital, Oujda, Morocco; Faculty of Medicine and Pharmacy, Laboratory of Anatomy, Microsurgery and Surgery Experimental and Medical Simulation (LAMCESM), Mohammed 1st University, Oujda, Morocco

**Keywords:** Cocaine, Colonic ischemia, Peritonitis, Surgery, Case report

## Abstract

**Introduction and importance:**

Cocaine, the second most abused drug in Morocco after cannabis, has been associated with multiple cardiac, pulmonary, neurological, and digestive complications. Colonic perforation following cocaine abuse is relatively lesser-known and requires attention as abuse rates are increasing, and existing evidence is scarce. Only a few cases have been reported in medical literature.

**Case presentation:**

We report the case of a 42-year-old male cocaine addict who presented with acute peritonitis. A laparotomy revealed a 3 cm perforation in the sigmoid, The absence of radiological, biological, and pathological evidence confirms the toxic origin of the perforation.

**Clinical discussion:**

Cocaine-induced ischemic colitis is a rare occurrence in a surgeon's clinical experience. This condition is typically confirmed through colonoscopy and often resolves without the need for surgery, although a small number of cases may advance to peritonitis, necessitating surgical intervention.

**Conclusion:**

Cocaine's adverse effects should be taken into account in the differential diagnosis of acute ischemic events in young adults. A general understanding of the significant complications associated with cocaine can aid in achieving early diagnosis and prompt treatment.

## Highlights

• Digestive perforation is a rare but serious complication of cocaine user.
• Peritonitis by spontaneous colonic perforation of the digestive tract is the most serious complication.
• The possible cocaine-related ischemic event is the cause of perforation in these patients.
• For acute abdominal pain in cocaine users, consider digestive perforation in the emergency department
• Early exploratory laparotomy may be the first-line treatment.
• Prevention is better than cure

## 1 Introduction

Cocaine, also known as benzoyl methyl ecgonine, is a crystalline tropane alkaloid derived from the leaves of the *Erythroxylum coca* plant. A granular crystalline powder, cocaine hydrochloride, which can be smoked, is produced by dissolving the alkaloid in hydrochloric acid [1].

In a survey conducted in June 2019 by the Moroccan Society of Clinical and Analytical Toxicology on drug, tobacco, and alcohol usage among the Moroccan population, it was found that among individuals using psychotropic drugs, 50 % preferred cannabis, while 12 % used cocaine, and 3 % used heroin [2].

Cocaine has long been a substance of abuse. Oral, inhalation, intravenous, and intranasal cocaine abuse has been associated with several surgical complications [3]. Colonic perforation, while rare, remains a severe manifestation that should be considered in all cocaine users.

In this report, we present a case involving sigmoidal complications leading to generalized peritonitis in a chronic crack cocaine user, in accordance with the updated guidelines for consensus-based surgical case reports (SCARE 2023) [4].

## 2 Case presentation

We admitted a 42-year-old patient to the surgical emergency department with severe abdominal pain intensified in the left iliac fossa evolving for 2 days, associated with vomiting without fever, or any other symptom of gastrointestinal obstruction. He had no significant medical history, including no prior constipation or tuberculosis infection. The patient admitted a daily crack cocaine use over the past year but denied experiencing chest pain or shortness of breath. He also mentioned smoking a pack of cigarettes daily and occasional alcohol consumption.

His vital signs were as follows: blood pressure 101/68 mmHg, pulse rate 80 beats/min, respiration rate 18 breaths/min, and a temperature of 37 °C. His oxygen saturation was 98 % on room air. The abdominal examination revealed generalized abdominal tenderness. A rectal examination did not reveal any blood or tenderness, and the rest of the examination was unremarkable. An abdominal X-ray did not show signs of pneumoperitoneum.

The patient's laboratory workup indicated hyperleukocytosis at 13,040/μl and a C-reactive protein level of 248 mg/l. Viral serology tests (hepatitis, HIV, EBV, and CMV) were ordered and showed no abnormalities. Various diagnostic tests were conducted, all of which returned negative results.

**Fig. 1:**
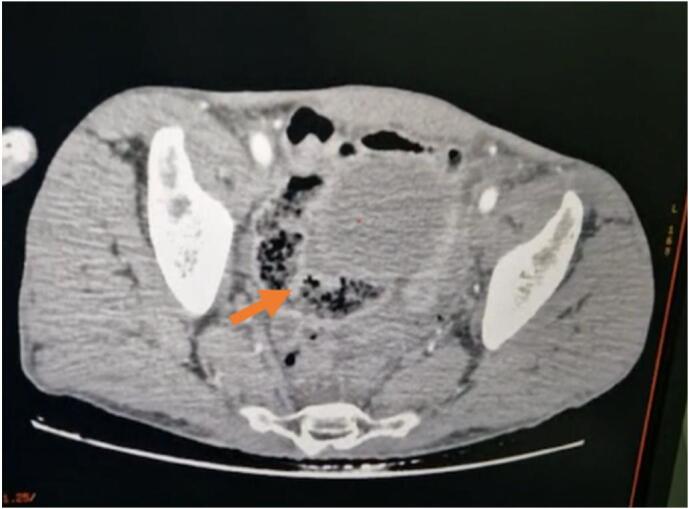
Image showing the presence of a pre-rectal pelvic collection, with stercoral content and thin wall enhanced after contrast injection measuring 68 * 31 mm.

**Fig. 2:**
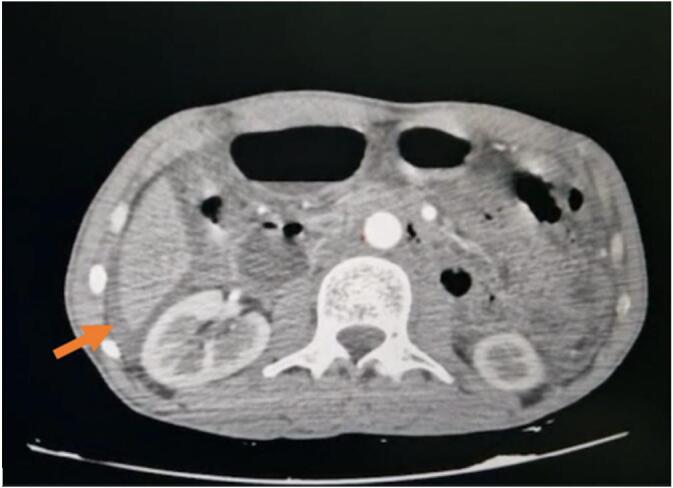
Intraperitoneal fluid effusion of great abundance, peri- and sub-hepatic, peri- and sub-splenic, inter-anal, in the parietal-colic gutters and intra-pelvic associated with enhancement of the peritoneal layers.

A computed tomography (CT scan) was recommended, which revealed generalized peritonitis with a pre-rectal pelvic collection (see Fig. 1, Fig. 2), containing stercoral content and extra-digestive air bubbles both above and below the mesocolic area.

**Fig. 3:**
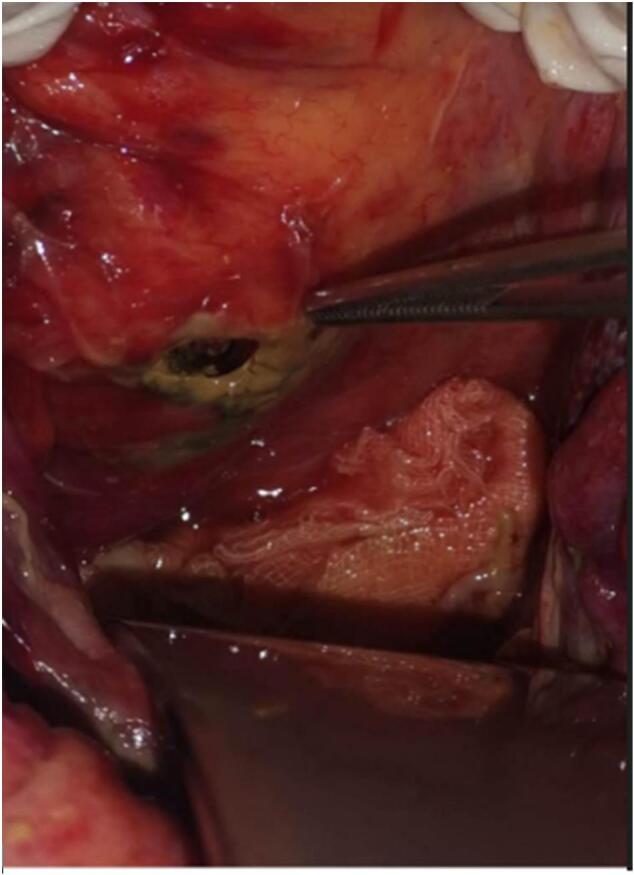
Image showing the presence of the sigmoidal perforation.

Following a discussion with the patient and obtaining his consent, an urgent exploratory laparotomy was performed. The procedure revealed a significant purulent effusion, indicating generalized peritonitis, with the identification of a sigmoid perforation measuring approximately 3 cm. An examination of the colonic framework did not reveal any colonic necrosis, diverticula or other inflammatory or tumoral lesions. Biopsies were taken from the edges of the perforation, and the sigmoid perforation was sutured, along with the creation of a stoma near the sigmoid colon upstream of the perforation due to the septic condition. Additionally, a drain was placed opposite the sutured perforation (refer to Fig. 3).

**Fig. 4:**
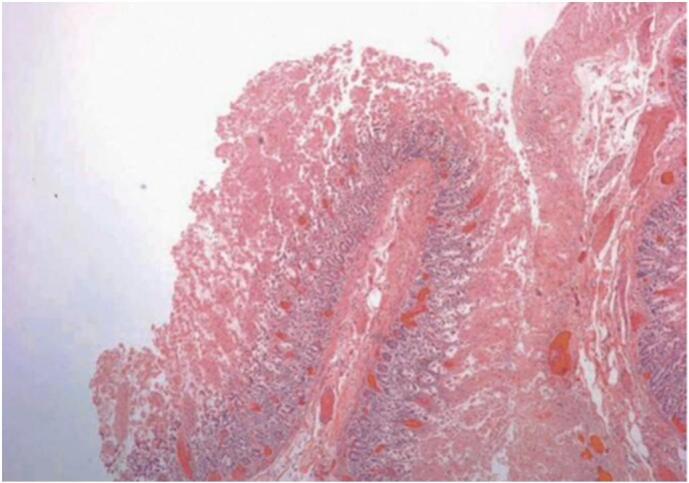
Microphotograph showing a resected mucosal hemorrhagic necrosis in ischemic colitis.

Pathological examination of the biopsy showed extensive ulceration of the mucosa with arteriolar and venous thrombosis and hemorrhage in the wall of the intestine (see Fig. 4).

The patient was discharged on the 4th day following improvement in his infectious workup. The patient returned for a surgical consultation on the 7th day after discharge, with no specific issues to report.

A total colonoscopy was performed three months postoperatively, revealing petechial or focal hemorrhage and mucosal edema in the transverse and ascending colon.

He was scheduled for an addiction consultation and to undergo the restoration of colonic continuity.

## 3 Discussion

Cocaine, derived from the leaves of the *Erythroxylum coca* plant, is a crystalline tropane alkaloid that exerts its central action by inhibiting the reuptake of dopamine, norepinephrine, and serotonin, leading to the activation of the central and sympathetic nervous systems. [1]. This stimulation can lead to intestinal and abdominal ischemia, although the exact mechanism of cocaine-induced ischemia is not fully defined. It is suggested that three causal factors may be involved in the development of intestinal ischemia: arteriolar vasospasm, platelet activation, and accelerated atherosclerosis.

The majority of literature on cocaine-related bowel ischemia consists of case reports and small case series, indicating that the mortality associated with cocaine-induced ischemia may be high. However, many studies lack control groups, and the sample sizes are often too small to draw definitive conclusions [5].

In our case, after excluding all other potential causes, it was determined that cocaine use was the primary factor contributing to the sigmoidal perforation.

The pathophysiology of cocaine-induced ischemia is relatively well understood. Cocaine inhibits the reuptake of norepinephrine at presynaptic terminals, leading to an accumulation of catecholamines at postsynaptic membranes. This influx of catecholamines results in tachycardia, vasoconstriction, and hypertension. Vasoconstriction can cause ischemia, which, in turn, affects multiple organ systems and may lead to mesenteric vasoconstriction and localized blood pressure elevation. This focal tissue ischemia is likely to result in perforation [6].

Cocaine has been found to have additional mechanisms that can contribute to intestinal ischemia. It exerts a direct vascular constrictive effect by increasing calcium flow across the endothelial cell membrane. Some have suggested that cocaine has a direct toxic impact on the intestinal mucosa. Cocaine use also induces thrombus formation and platelet aggregation and reduces tissue fibrinolysis. Furthermore, it decreases fibrinolytic activity by stimulating plasminogen activator inhibitor activity. Some or all of these effects may explain the increased vulnerability of the colon to ischemia in individuals who abuse this drug [7].

The global incidence of gastrointestinal complications due to cocaine use is not well-documented. However, in one series from the USA, a single hospital treated 50 patients with juxtapyloric perforation over four years. This series reported only gastroduodenal perforation, and it is unclear how many other patients were admitted with different gastrointestinal complications resulting from drug abuse [8]. In Morocco, the number of reported cases is limited, although it is expected to increase with the growing number of cocaine users, which is explained in the United States by its geographical proximity to the countries producing this illicit drug [8].

Colonic perforations associated with cocaine smoking have been documented and are believed to result from deep colonic ulcerations caused by the multisystemic toxicity of freebase crack [9,10]. For example, Kram et al. reported four patients with perforated colonic ulcers due to crack use [11], and Kodali and Gordon reported upper gastrointestinal bleeding secondary to crack use [12]. Cocaine's blockade of norepinephrine reuptake leads to mesenteric vasoconstriction and focal tissue ischemia, which more frequently results in perforation [10,13].

In our case, the patient presented with abdominal pain and anorexia but without gastrointestinal hemorrhage. A CT scan revealed peritonitis due to sigmoidal perforation and inflammatory thickening of the left colon. In a study by Elramah et al., the most common symptoms included mucosal edema, pallor, and pneumatosis of the colonic wall, with thickening of the colonic wall being the most common CT finding. The prevalence of ischemia in different colon segments was as follows [5]:

• Ischemia on the left side of the colon was observed in 7 out of 78 patients.
• Ischemia on the right side of the colon was observed in 24 out of the 78 patients.

Cocaine-induced ischemic colitis may be an uncommon occurrence in a surgeon's clinical practice. The key point is to consider it as a potential diagnosis in patients presenting to the emergency department with acute abdominal pain, with or without rectal bleeding. Ischemic colitis is typically diagnosed through colonoscopy and often resolves without the need for surgery. However, in a few cases, it may progress to peritonitis, necessitating surgical intervention [14]. In our case, endoscopic examination was scheduled after the acute phase of peritonitis.

Elramah et al. couldn't determine whether a primary anastomosis or a colostomy should be performed as the initial surgical procedure [5]. In Chile, a right hemicolectomy was performed for toxic colonic ischemia with a cecum perforation [15]. In our case, We opted for suturing the perforation, creating a colostomy near the sigmoid colon upstream of the perforation, and performing lavage with drainage due to the patient's peritonitis.

## 4 Conclusion

Given the increase in cocaine and crack abuse in this country, surgeons need to be aware of their abdominal complications, especially mesenteric ischemia and gastroduodenal perforation, which mainly affect the younger age groups. These conditions should always be considered in patients with a history of cocaine abuse and abdominal pain to avoid delays in diagnosis and treatment. Cocaine addiction should be prevented and managed at the detoxification center.

## Consent

Written informed consent was obtained from the patient for publication of this case report and accompanying images. A copy of the written consent is available for review by the Editor-in Chief of this journal on request.

## Ethical approval

It's a one case report needing no ethnical approval.

## Funding

No sources of funding to our research has been needed.

## Author contribution

**Mabrouk Mohamed Yassine**: Writing, review, editing of the manuscript and participated in the surgery.

**Abdelali Guellil**: have helped writing the article, data collection.

**Soussan Haitam, Tarik Deflaoui**: Contributed for diagnose and treatment of the patient.

**Jabi Rachid**: supervised the writing of manuscript.

**Bouziane Mohammed** (oncology surgery professor): have supervised the writing of the.

Paper.

## Guarantor

Mabrouk Mohamed Yassine.

## Research registration number

Our paper is a case report; no registration was done for it.

## Conflict of interest statement

The authors declared no potential conflicts of interests with respect to research, authorship and/or publication of the article.
